# *Anaplasma platys* in Dogs, Chile

**DOI:** 10.3201/eid1309.070021

**Published:** 2007-09

**Authors:** Katia Abarca, Javier López, Cecilia Perret, Javier Guerrero, Paula Godoy, Ana Veloz, Fernando Valiente-Echeverría, Ursula León, Constanza Gutjahr, Teresa Azócar

**Affiliations:** * Pontificia Universidad Católica de Chile, Santiago, Chile; † Faculty of Veterinary Medicine, Universidad Santo Tomás, Santiago, Chile; ‡ Alcántara Veterinary Clinic, Santiago, Chile

**Keywords:** Anaplasma platys, anaplasmosis, ehrlichiosis, dog diseases, tick-borne diseases, polymerase chain reaction, 16S rRNA, groESL operon, phylogenetic analysis, dispatch

## Abstract

We conducted a 16S rRNA nested PCR for the genus *Ehrlichia* and *Ehrlichia spp.* with blood samples from 30 ill dogs in Chile. Phylogenetic analysis was performed by using *groESL* gene amplification. We identified *Anaplasma platys* as 1 of the etiologic agents of canine ehrlichiosis.

Ehrlichioses are recognized as important emerging tickborne diseases in humans and wild and domestic animals. The brown dog tick, *Rhipicephalus sanguineus*, is the main tick that infests dogs in Chile (*1*). This tick species is a vector of *Ehrlichia canis* and has been implicated, but not confirmed, as a vector of *Anaplasma platys* (*2*). Serologic and clinical evidence of canine ehrlichiosis and serologic evidence of human ehrlichiosis have been reported in Chile (*3**,**4*). The purpose of this study was to identify the etiologic agent of canine ehrlichiosis in Chile.

## The Study

Blood samples were obtained from 30 pet dogs seen in a private veterinary clinic in Santiago, Chile, with tick infestation and clinical signs compatible with ehrlichiosis (hemorrhagic manifestations and thrombocytopenia). We performed a nested PCR to amplify a portion of the 16S rRNA gene by using specific primers for the genus *Ehrlichia* and for *Ehrlichia* spp. DNA was extracted from 300 μL of whole blood by using the Wizard Genomic DNA Purification kit (Promega, Madison, WI, USA). For *Ehrlichia* genus–specific PCR, 2.5 μL of DNA was amplified by using outer primers EHR-OUT1 and EHR-OUT2 and inner primers GE2F and EHRL3-IP2 in 1 reaction with a final volume of 25 μL (*5*) (Table 1).

**Table 1:** *Ehrlichia/Anaplasma* spp. PCR primers used in this study

*Ehrlichia/Anaplasma* spp. (primer type)	Primer	Primer sequence (5′→3′)	Region	Reference
*Ehrlichia* spp., *A. phagocytophilum*, *E. canis*, *E. chaffeensis*, *E. ewingii, A. platys* (outer)	EHR-OUT1	CTGGCGGCAAGCCTAACACATGCCAACAT	16S rRNA	(*5*)
	EHR-OUT2	GCTCGTTGCGGGACTTAACCCAACATCTCACGAC	16S rRNA	(*5*)
*Ehrlichia* spp. (inner)	GE2F	GTTAGTGGCATACGGGTGAAT	16S rRNA	(*5*)
	EHRL3-IP2	TCATCTAATAGCGATAAATC	16S rRNA	(*5*)
*A. phagocytophilum* (inner)	ge9f	AACGGATTATTCTTTATAGCTTGCT	16S rRNA	(*6*)
	ge2	GGCAGTATTAAAAGCAGCTCCAGG	16S rRNA	(*6*)
*E. canis*, *E. chaffeensis*, *E. ewingii* (inner)	HE3-R	CTTCTATAGGTACCGTCATTATCTTCCCTAT	16S rRNA	(*5*)
*E. canis* (inner)	*E. canis*	CAATTATTTATAGCCTCTGGCTATAGGAA	16S rRNA	(*5*)
*E. chaffeensis* (inner)	*E. chaffeensis*	CAATTGCTTATAACCTTTTGGTTATAAATA	16S rRNA	(*5*)
*E. ewingii* (inner)	*E. ewingii*	CAATTCCTAAATAGTCTCTGACTATT	16S rRNA	(*5*)
*E. equi* (inner)	*E. equi-3-IP2*	GTCGAACGGATTATTCTTTATAGCTTG	16S rRNA	(*5*)
*E. platys* (inner)	*EHRL3-IP2*	TCATCTAATAGCGATAAATC	16S rRNA	(*5*,*7*)
	*E. platys*	GATTTTTGTCGTAGCTTGCTA	16S rRNA	(*7*)
*E. platys* (outer)	EEgro1F	GAGTTCGACGGTAAGAAGTTCA	*groESL*	(*8*)
	EEgro2R	CAGCGTCGTTCTTACTAGGAAC	*groESL*	(*8*)
*A. platys* (inner)	SQ3F	ATTAGCAAGCCTTATGGGTC	*groESL*	(*9*)
	SQ5F	TCAGTGTGTGAAGGAAGTTG	*groESL*	(*9*)
	SQ4R	CTTTAGGCTATCAAGAGATG	*groESL*	(*9*)
	SQ6R	TGCTTCCTATGTTCTTATCG	*groESL*	(*9*)

The first-round amplification included 20 cycles of denaturation at 94°C for 45 s, annealing at 72°C for 1.5 min, and chain extension at 72°C for 1.5 min. The second-round amplification included 50 cycles of denaturation at 94°C for 45 s, annealing at 50°C for 1 min, and chain extension at 72°C for 1 min, followed by a final extension at 72°C for 5 min. Amplification products were analyzed by agarose gel electrophoresis. The expected size of the amplification product was 120 bp. *A*. *phagocytophilum* DNA was used as a positive control (provided by Didier Raoult). For *Ehrlichia* spp.–specific amplification, we used the same set of outer primers for *Anaplasmataceae* and specific inner primers for *A*. *phagocytophilum* (*6*), *E*. *chaffeensis*, *E*. *ewingii*, and *E*. *canis* (*5*) (Table 1). For *A*. *platys* amplification, we used inner primers developed by Kordick et al. (EHRL3-IP2–*E. platys*) (*7*) (Table 1). Expected sizes of amplification products were 546, 395, 395, 389, and 151 bp, respectively.

**Figure 1:**
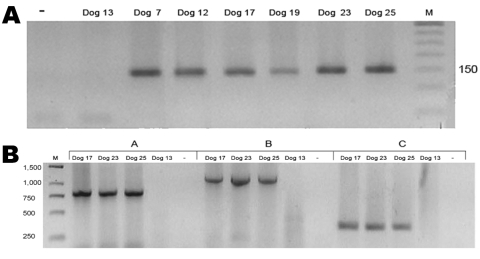
A) *Anaplasma platys* nested PCR products of 30 blood samples from dogs in Chile. Positive samples from dogs 7, 12, 17, 19, 23, and 25 are indicated by a 150-bp band. –, PCR-negative control; dog 13, negative control; M, 50-bp DNA ladder. Value on the right is in basepairs. B) Second-round *A. platys groESL* nested PCR products of dog DNA samples with 3 sets of primers. Group A, SQ5F/SQ4R (790 bp); group B, SQ3F/SQ4R (1,170 bp); group C, SQ3F/SQ6R (360 bp). M, GeneRuler 1-kb DNA ladder (Fermentas, Hanover, MD, USA); Dog 13, negative control; –, PCR-negative control. Values on the left are in basepairs.

The *Ehrlichia* genus PCR resulted in the expected DNA band in 6 of 30 dogs (dogs 7, 12, 17, 19, 23, and 25). These 6 samples were positive only for *A. platys*, showing the expected 151-bp product, and negative for other species tested (Figure 1, panel A). *A*. *platys* PCR was also conducted on the remaining 24 *Ehrlichia*-negative samples; none were positive.

DNA obtained from 3 16S rRNA PCR products (dogs 7, 17, and 25) was purified by using a commercial kit (Rapid Gel Extraction System; Marligen Biosciences, Ljamsville, Germany) and sequenced twice with an ABI 3100 genetic analyzer (Model 3100; Applied Biosystems, Foster City, CA, USA). The 16S rRNA sequences obtained were compared by using BLAST (www.ncbi.nlm.nih.gov/blast) with sequences available at GenBank. Sequences obtained were similar to that of *A*. *platys* strain Okinawa 1 (GenBank accession no. AF536828), with similarities of 98%, 95%, and 98%, respectively. GenBank accession nos. for 16S rRNA sequences of *A*. *platys* strains obtained in this study are DQ125260 and DQ125261, which correspond to strains from dogs 7 and 17, respectively.

For phylogenetic analysis, the *groESL* gene of *A*. *platys* was amplified from samples positive for *A*. *platys* 16S rRNA that had sufficient amounts of DNA (dogs 17, 23, and 25) and from 1 negative sample (dog 13). Reactions contained 2 μL of purified DNA as template in a total volume of 25 μL. Amplifications contained 1.25 U *Taq* DNA polymerase (Invitrogen, Carlsbad, CA, USA), 3 mmol/L MgCl_2_, 2.5 mmol/L deoxynucleotide triphosphates (Invitrogen), and 0.2 pmol/L of primers EEgro1F and EEgro2R (*8*) (Table 1). DNA was denatured by heating at 95°C for 10 min. PCR amplification included 40 cycles of denaturation at 95°C for 1.5 min, annealing at 52°C for 2 min, and extension at 72°C for 1.5 min, followed by a final extension at 72°C for 10 min. For nested amplifications, 1 μL of primary PCR products was used as the template in a total volume of 25 μL. Reaction conditions were the same as for primary amplifications. The primers used were SQ3F, SQ5F, SQ4R, and SQ6R (*9*) (Table 1). PCR products were analyzed by 1.5% agarose gel electrophoresis.

**Figure 2:**
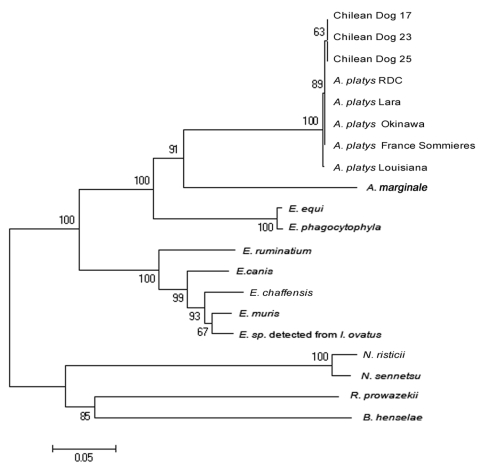
Phylogenetic relationship between 3 Chilean *Anaplasma platys* strains and other strains of the families *Rickettsiaceae* and *Anaplasmataceae* based on the *groESL* gene nucleotide sequences. GenBank accession nos. of *groESL* sequences used to construct the phylogenetic tree were the following: *A. platys* France Sommieres AY044161*; A. platys* Lara Venezuelan dog AF399916; *A. platys* from *Rhipicephalus sanguineus* ticks in the Democratic Republic of Congo AF478129; *A. platys* from a dog in Okinawa, Japan AY077621; *A. platys* from a dog in Louisiana, USA AY008300; *A. marginale* AF165812; *Ehrlichia equi* AF172162; *E. phagocytophyla* U96729*; E. chaffeensis* L10917; *E. canis* U96731; *E. muris* AF210459; *Ehrlichia* sp. from *Ixodes ovatus* AB032711; *E. ruminantium* U13638; *Neorickettsia risticii* U96732; *N. sennetsu* U88092; *Rickettsia prowazekii* Y15783; and *Bartonella henselae* U96734. Scale bar at the lower left indicates 0.05 substitutions per nucleotide.

We amplified 3 overlapping fragments (790, 1,170, and 360 bp) in 3 16S rRNA–positive samples (Figure 1, panel B). These DNAs were purified by using a commercial kit (Rapid Gel Extraction System; Marligen), sequenced, and analyzed for phylogenetic relationships. Multiple alignment analysis was performed with the ClustalW program (www.ebi.ac.uk/clustalw). Calculation of distance matrices and construction of a phylogenetic tree were made with MEGA 3.1 software (www.megasoftware.net). A phylogenetic tree was constructed by the neighbor-joining method and distance matrices for the aligned sequences were calculated by using the Kimura 2-parameter method. Stability of the tree was estimated by bootstrap analysis of 1,000 replications. A final sequence of 686 bp obtained from the overlapping fragments was used for comparison and showed 100% identity between the 3 Chilean sequences and 99.8% similarity with sequences of the *A*. *platys groESL* gene deposited in GenBank (Table 2). Phylogenetic relationships of Chilean *A*. *platys* strains with other *Anaplasmataceae* species are shown in Figure 2. GenBank accession no. for the *groESL* gene sequence of *A*. *platys* is EF201806 (corresponding to dogs 17, 23, and 25).

**Table 2:** Nucleotide sequence differences among *groESL* genes from different strains of *Anaplasma platys*

Strain	Similarity,* %	Nucleotide position†		
591	1259	1271
*A. platys* Sommieres	100	G	A	C
*A. platys* Lara	100	–	–	–
*A. platys* RDC	100	–	–	–
*A. platys* Okinawa	100	–	–	–
*A. platys* Louisiana	99.7	–	G	T
Dog 17	99.8	T	–	–
Dog 23	99.8	T	–	–
Dog 25	99.8	T	–	–

*Percentages of nucleotide sequence identities for 686-bp region determined from pairwise alignment. †Nucleotide positions of *A. platys* Sommieres strain, GenBank accession no. AY0441621. –, same base as the type strain.

## Conclusions

We identified *A*. *platys* DNA in the blood of 6 dogs with clinical signs indicative of ehrlichiosis. These findings support the conclusion that *A*. *platys* is an etiologic agent of canine ehrlichiosis in Chile.

Since its first report in the United States in 1978 (*10*), *A*. *platys* has been described in several countries as the etiologic agent of cyclic thrombocytopenia in dogs. A tick vector of *A*. *platys* has not been determined, although *R*. *sanguineus* is the most suspected species (*2*). Because *R*. *sanguineus* is the only tick species that infests dogs in Santiago (*1*), our results support the conclusion that this species is the vector of *A*. *platys* in Chile.

A wide range of clinical manifestations of canine cyclic thrombocytopenia has been described. Cases from the United States have been described as mild or asymptomatic (*10*), and cases from Spain have more severe symptoms (*11*), which also seems to be the case in Chile. This variability in clinical symptoms of infection has not been clearly associated with strain variations (*11*–*13*).

Low diversity was observed when *groESL* gene sequences of Chilean strains were compared with other *A*. *platys* strains available in GenBank. This finding has also been observed in strains from different geographic origins (*13*). Recent studies have shown more genetic variability when sequences of the *gltA* gene were used (*11*,*12*).

Evidence of the zoonotic potential of *A*. *platys* is scarce. In Venezuela, a few symptomatic human cases have been diagnosed since 1992 by the presence of platelet morulae in blood smears (*14*). Monocytic and platelet morulae were reported in a 17-month-old girl with fever and rash (*15*). However, none of these cases have been confirmed by molecular assays. Further studies that investigate the pathogenic and zoonotic role of *A*. *platys* should be conducted.
